# Use of CD-ROM MEDLINE by Medical Students of the College of Medicine, University of Lagos, Nigeria

**DOI:** 10.2196/jmir.5.1.e7

**Published:** 2003-03-31

**Authors:** Taiwo O Ogunyade, Wellington A Oyibo

**Affiliations:** ^1^University of LagosDepartment of Medical Microbiology and ParasitologyCollege of MedicineLagosNigeria

**Keywords:** MEDLINE use, students, medical, MEDLINE search, MEDLINE assignment, MEDLINE service, Nigeria, information retrieval, medical library, libraries, medical

## Abstract

**Background:**

Use of information technology in information acquisition, especially MEDLINE on CD-ROM and online, has been evaluated in several localities and regions, especially in the advanced countries. Use of MEDLINE on CD-ROM is still very poor among the medical students of the University of Lagos, Lagos, Nigeria, due to lack of awareness, insufficient personal computers, nonperiodic training, and the high cost of using the facility. Due to financial constraints, MEDLINE online and sufficiently-networked computer systems are not available.

**Objective:**

To report on the situation in Nigeria, a developing country, so as to compare the current awareness of searching MEDLINE on CD-ROM among the medical students at the University of Lagos with the awareness of their overseas' counterparts. This is the first step toward setting up an online PubMed search as well as expanding the computer systems and network.

**Methods:**

Essentially based on cross-sectional proportional sampling using structured questionnaires, in-depth interviews, and focus-group discussions among the medical students and library staff. The study involved the medical students in their second year to sixth (final) year of study.

**Results:**

Of the 250 students interviewed, 130 (52%) were aware of MEDLINE on CD-ROM searches as a means of information retrieval. Only 60 (24%) had used MEDLINE on CD-ROM — 2% had used MEDLINE on CD-ROM more than 9 times; 4%, 7 to 9 times; 8%, 4 to 6 times; and 10%, 1 to 3 times. Of the students who used MEDLINE on CD-ROM search, 22% used it in preparing for examinations, 24% in research, 6% in patient care, and 26% in preparation of assignments and clinical cases. Lack of awareness (52%) and cost of undertaking MEDLINE on CD-ROM search (46%) were identified as important factors that discouraged the use of MEDLINE on CD-ROM.

**Conclusions:**

Though the above factors were recognized as important, it was concluded that the reasons for the poor use of MEDLINE on CD-ROM are multifactorial. Poor use of MEDLINE on CD-ROM could be attributed to these critical underlying factors: nonavailability of networked personal computers, which should be connected to a central server; lack of mandatory assignments to the medical students that would specifically require use of MEDLINE on CD-ROM; financial constraints on the university management; and infrequent periodic orientation on use of MEDLINE on CD-ROM. It was therefore suggested that the number of personal computers should be increased and that the library staff should periodically train the preclinical and clinical medical students in searching MEDLINE on CD-ROM. These steps would enable the medical students to benefit from online PubMed searching when it becomes fully operational in the future.

## Introduction

The application of information technology has revolutionized the mode of acquisition, storage, and dissemination of information. Today, computer access to the medical literature has become more convenient than ever before with MEDLINE on CD-ROM and online. Computerized literature searching enables the user to efficiently identify germane articles and research studies. Physicians can also learn to use a computerized literature-searching system and efficiently retrieve materials related to patient care [1,2]. In medical education, the literature also functions as source material for conference presentations, course and licensing examinations, teaching assignments, and research studies [3- 5]. While educators agree that medical students should learn to use MEDLINE for clinical application, there is a lack of consensus on an optimal level of exposure to this resource during training that will result in sustained usage [6].

Although the Medical Library of the College of Medicine of the University of Lagos, Lagos, Nigeria, subscribes to a considerable number of worldwide medical journals, the unavailability of these journals when they are supposed to be available impedes the supply of information [7]. The problem of supply of current journals is due largely to cost, and this is where MEDLINE on CD-ROM becomes useful in the provision of primary information pending the arrival of the journals.

Online MEDLINE facilitates the retrieval of information from over 4500 journals published throughout the world. The availability of the full text of publications and articles implies that this process, with its recognizable advantages, will be useful in the training of medical students. Over the years, the Medical Library of the University of Lagos has made efforts toward providing MEDLINE both on CD-ROM and online in addition to other facilities that would enhance information acquisition. However, lack of funds to purchase the required computers and to network the computers with online connections limited these efforts to the provision of MEDLINE on CD-ROMs that do not require online connection. Since the personal computers are few, networking was also not available. The extent to which the medical students utilize the available facilities for MEDLINE on CD-ROM in their training is not known. We therefore investigated the awareness and use of MEDLINE on CD-ROM by the medical students of the University of Lagos, College of Medicine in comparison with what exists overseas. This is the first step toward the introduction of online PubMed search when funds become available.

## Methods

### Study Area

The Medical Library of the College of Medicine, University of Lagos, was used for this study. This library serves the entire medical community made up of undergraduate (medical, dental, pharmacy, and basic medical sciences) and postgraduate students (MSc, MPhil, and PhD levels), resident doctors, nurses, medical laboratory technologists, health workers, and the academics. Generally, the medical library serves the Lagos University Teaching Hospital (LUTH), the School of Nursing and Midwifery, Post-Basic Nursing, the School of Medical Laboratory Technology, and the School of Medical Records and Statistics.

The College of Medicine of the University of Lagos is made up of: the School of Basic Medical Sciences, the School of Clinical Sciences, the School of Dental Sciences, the School of Pharmaceutical Sciences, and the Institute of Child Health and Primary Health Care. Only the medical students enrolled for the Bachelor of Medicine and Surgery (MBBS) or the MD program were used in this study.

The Medical Library usually conducts a one-week orientation program for newly-enrolled medical students. During this period, the students are trained in effective ways of using the library, including using MEDLINE on CD-ROM.

### Study Group

The study was conducted between June and September 2002 among the medical students of the College of Medicine, University of Lagos. The randomly-selected medical students were in the second year to sixth (final) year. The premed class was not studied, because the students were located in another campus and were yet to be enrolled in medical school.

The sampling size of this study was 230 students. A proportional sampling unit was selected from each class to ensure unbiased representation. For this study, 340 questionnaires were administered. Structured questionnaires were administered to each student. This was followed by focus-group discussions and in-depth interviews with key informants among the studied population. The focus group and key informants were randomly selected from the student population. Information was also obtained from designated class representatives of the students and library staff. Clarification that the study was not intended to punish non-users of MEDLINE on CD-ROM — the names of the students were not required in the questionnaire — enhanced participation. This step removed bias from the responses provided by the medical students.

## Results

Of the 340 questionnaires that were administered, 250 were duly completed. This represented a response rate of 73.5%. The sample size of 230, which was used in the study, could be taken as 100% response in principle. The study selected: 40 students in the sixth year class (16%); 65 in the fifth year class (26%), 25 in the fourth year class (10%); 90 in the third year class (36%) and 30 in the second year class (12%).

### Interview Results

Of the 250 students interviewed about MEDLINE on CD-ROM: 130 (52%) were aware of it, while 48% did not know what it was about; 120 (48%) were aware it was provided by the medical library; 60 (24%) had used it at one time or another, while 190 (76%) had not used it.

The frequency of use of MEDLINE on CD-ROM was: 1 to 3 occasions, 10%; 4 to 6 occasions, 8%; 7 to 9 occasions, 4%; and more than 9 occasions, 2% (percentages are based on the 250 students interviewed; percentages sum to the 24% that had used MEDLINE on CD-ROM at one time or another).

Of the 60 students who had used MEDLINE on CD-ROM at one time or the other, their purposes were: preparing for examination, 22%; personal interest, 20%; research, 24%; patient care, 6%; and preparation of assignments or clinical cases, 26% (percentages are based on the 60 students who had used MEDLINE on CD-ROM).

Of the 250 medical students interviewed, 40 (16%) stated that MEDLINE on CD-ROM was very useful; 20 (8%) useful, and 15 (6%) fairly useful.

Lack of awareness and the cost of MEDLINE on CD-ROM searching were the major factors responsible for its poor use (Table 1). The majority of the respondents who had used MEDLINE on CD-ROM printed copies of abstracts. The students who had used MEDLINE on CD-ROM would prefer to use it again because it saves time while giving them the opportunity to conduct as many searches as possible within a short time.

**Table 1:** Factors responsible for the poor use of MEDLINE facilities among medical students of the College of Medicine, University of Lagos, Nigeria

**Factor**	**Number (%)**
Lack of awareness	130 (52)
Cost of using MEDLINE	115 (46)
Inconvenient location of the MEDLINE facility	20 (8)
Inconvenient hours of undertaking search	15 (6)
Service of MEDLINE not needed	5 (2)
Waiting period for undertaking the search	5 (2)

### Focus Group Results

Focus Group discussions and interview of key informants exposed 3 issues:

#### Lack of Awareness Due to Non-emphasis on MEDLINE

Examples of responses:

- "We were always told to look up journals in the journal section for new information"
- "We have never been told to use MEDLINE; any way, there is so much to read in our text-books"
- "We are used to hard copies of texts and journals"
- "Though we received some orientation on how best to use the library during our first year of enrolment in the medical school, the trend has always been the use of text-books and journals and attend clinics regularly to read case notes"
- "MEDLINE is needed by the postgraduate students and resident doctors because they are doing serious research"

#### Cost of undertaking MEDLINE search on CD-ROM

They agreed that "the cost and waiting time, which could take several days if a particular class would have to seek information in a given time, made us to always use the available journals and then pay for photocopies." The waiting time is due to the limited number of personal computers, which are not networked.

#### Insufficient Personal Computers and Lack of Periodic Orientation

Examples of responses:

- "Access is somewhat restricted because of the limited number of PCs; thus, we the undergraduate will have to compete for the available computers with the Postgraduate students".
- "We will be happy to have periodic orientation on MEDLINE use as well as having more computers to make the training qualitative"

## Discussion

The advent of information technology, including the use of MEDLINE facilities, has, over the years, facilitated the acquisition of information. This is diametrically different from manual searches using Index Medicus and other bibliographies. To a large extent, MEDLINE (CD-ROM and online) is fully integrated into the university system in developed countries but is yet to be fully utilized in developing countries. The reasons for this poor utilization range from structural inadequacies to lack of awareness.

The usefulness of MEDLINE on CD-ROM and online are obvious and have been variously reported, though they differ from one institution to another [3- 5,8,9]. Information acquisition is therefore difficult in developing countries where the referred journals are not available, thus making it imperative for universities to acquire new technologies such as having a highly-networked computer system that has online connections [7].

The results of this study showed that use of MEDLINE on CD-ROM is poor among the medical students. The reasons for this poor use are multifactorial. First is the attitude of the students toward MEDLINE CD-ROM use — which has made reorientation difficult. In-depth interviews revealed that the students, in most cases, are contented with printed materials available on the shelf. The students' use of printed materials is strengthened by the use of texts by students in the preclinical class (first and second year) in preparing for examinations (including in-course assessments). Our findings on use of MEDLINE on CD-ROM therefore differed from the 87% use of the computerized literature-searching system for patient write-ups by medical students of the Wright State University School of Medicine (WSUSOM), Ohio, USA [2]. Unlike the system in WSUSOM that enabled the user to have the full text of a journal article or textbook in addition to citations and abstract [2], our system only provides abstracts. The students interviewed considered abstracts inadequate.

However, the factors responsible for poor use of MEDLINE CD-ROM (Table 1) are important and crucial. Most important of these factors were lack of awareness and the cost of undertaking searches of MEDLINE on CD-ROM. This finding is consistent with the recognized factors found to discourage database use: cost, availability, and awareness [10]. Though these factors were theoretically not present in the study situation at the Pritzker School of Medicine of the University of Chicago, Illinois, USA, Kaluzsa [10] reported that all 3 factors still discouraged MEDLINE use for a significant portion of the student body. The results from our study were different when compared to what was obtained among students enrolled in the undergraduate program of the McMaster University Faculty of Health Sciences, Canada [11]. In this study — which was on the knowledge and use of information technology (competence, access, usage, and perceptions of the need for training) — there was a progressive rise in reported information technology access and use for 3 years. Self-service MEDLINE use on CD-ROM was 65%, 75%, and 89% for the 1987, 1988, and 1989 classes respectively [11].

Our study was also different from the results obtained by Osman and Muir [12] among 144 third-year students at the University of Edinburgh, Scotland, UK They reported that 22% of the students had never used the university-library computerized catalog and 43% had never carried out a MEDLINE search using the library's CD-ROM. This study in the UK [12] also asserted that medical students who have not acquired basic computer information-technology skills by the third year of undergraduate training are unlikely to do so in the final hospital-based years. In our study, 90 (36%) of the sampled group were from the third year class and only 12 (13.3%) of those 90 had used MEDLINE on CD-ROM.

It costs about US $0.11 to print a copy of an abstract. However, viewing an abstract does not require any form of payment. Even so, viewing would be difficult because the systems are not sufficient to serve those who would wish to pay for a literature search. The nonavailability of networked personal computers was identified during in-depth discussion with key informants as a critical structural inadequacy that is responsible for the nonuse of MEDLINE in the university. Furthermore, lack of awareness appears to be an important factor in the nonuse of MEDLINE.

In most cases, it is sufficient for the students to use the available materials from textbooks and journals for their assignments, patient care, case studies, etc. This can be explained by the materials being within the immediate reach of the students. Information technology is yet to be fully appreciated, with many students preferring to go the old way. Nevertheless, availability and access to software and personal computers remains a major issue, because even the faculty does not have easy access.

An important method of creating awareness is having the faculty require students do a MEDLINE search for information. It has been reported that connecting assignments and other academic activities to MEDLINE searching brought about an increase in the awareness and use of MEDLINE [2]. The organization of periodic training programs in the use of computerized information searching systems by the library and information-science staff would prove useful. For example, a short formal course of instruction in computer skills (computer basics, e-mail management, and MEDLINE and Internet search tools) organized for first-year medical students entering the State University at Stony Brook, New York, USA, was successful in achieving an acceptable level of comfort in using a computer for almost all of the student body [13].
